# Conformational heterogeneity of the Roc domains in *C. tepidum* Roc–COR and implications for human LRRK2 Parkinson mutations

**DOI:** 10.1042/BSR20150128

**Published:** 2015-10-08

**Authors:** Katharina Rudi, Franz Y. Ho, Bernd K. Gilsbach, Henderikus Pots, Alfred Wittinghofer, Arjan Kortholt, Johann P. Klare

**Affiliations:** * Department of Physics, University of Osnabrueck, Barbarastr. 7, 49076 Osnabrueck, Germany; † Department of Biochemistry, University of Groningen, Nijenborgh 4, Groningen 9747 AG, Netherlands; ‡ Department of Cell Biochemistry, University of Groningen, Nijenborgh 7, Groningen 9747 AG, Netherlands; § Structural Biology Group, Max Planck-Institute for Molecular Physiology, Otto-Hahn-Str. 11, 44227 Dortmund, Germany

**Keywords:** conformational heterogeneity, double electron–electron resonance (DEER), electron paramagnetic resonance (EPR) spectroscopy, G-protein, leucine-rich repeat kinase 2 (LRRK2), Parkinson's disease, Ras of complex proteins (Roc) domain, RocCOR tandem, Roco, structure

## Abstract

Ras of complex proteins (Roc) is a Ras-like GTP-binding domain that always occurs in tandem with the C-terminal of Roc (COR) domain and is found in bacteria, plants and animals. Recently, it has been shown that Roco proteins belong to the family of G-proteins activated by nucleotide (nt)-dependent dimerization (GADs). We investigated the RocCOR tandem from the bacteria *Chlorobium tepidum* with site-directed spin labelling and pulse EPR distance measurements to follow conformational changes during the Roco G-protein cycle. Our results confirm that the COR domains are a stable dimerization device serving as a scaffold for the Roc domains that, in contrast, are structurally heterogeneous and dynamic entities. Contrary to other GAD proteins, we observed only minor structural alterations upon binding and hydrolysis of GTP, indicating significant mechanistic variations within this protein class. Mutations in the most prominent member of the Roco family of proteins, leucine-rich repeat (LRR) kinase 2 (LRRK2), are the most frequent cause of late-onset Parkinson's disease (PD). Using a stable recombinant LRRK2 Roc-COR-kinase fragment we obtained detailed kinetic data for the G-protein cycle. Our data confirmed that dimerization is essential for efficient GTP hydrolysis and PD mutations in the Roc domain result in decreased GTPase activity. Previous data have shown that these LRRK2 PD-mutations are located in the interface between Roc and COR. Importantly, analogous mutations in the conserved *C. tepidum* Roc/COR interface significantly influence the structure and nt-induced conformational changes of the Roc domains.

## Abstract (precis)

Kinetic data for leucine-rich repeat (LRR) kinase 2 (LRRK2) confirms that dimerization is essential for efficient GTP hydrolysis and that Parkinson's disease (PD) mutations cause decreased activity. Investigation of the *Chlorobium tepidum* RocCOR tandem reveals conformational heterogeneity of the Roc domains and the influence of LRRK2-analogous PD-mutations.

## INTRODUCTION

**Figure 1:**
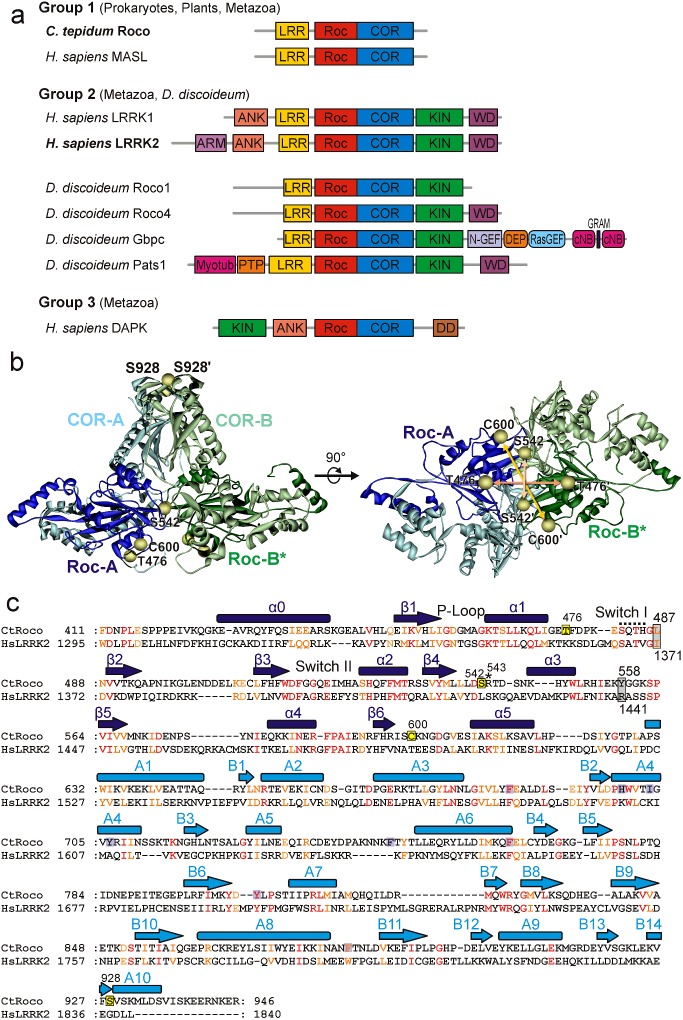
The Roco protein family (**a**) Domain topology of the Roco family proteins. The domains are ankyrin repeats (ANK), armadillo repeats (ARM), cyclic nt-binding domain (cNB), COR, death domain (DD), dishevelled, egl–10 and pleckstrin (DEP), Rab-like GTPase activators and myotubularins (GRAM), LRR, kinase (KIN), N-terminal motif of RasGEF (N-GEF), protein tyrosine phosphatase (PTP), RasGEF and Roc. (**b**) Model of the RocCOR dimer in two different orientations separated by 90°, with residues replaced by cysteine (except for Cys^600^) and subsequently labelled with MTSSL marked by spheres at the positions of their Cα atoms. The different protomers are shown in blue (light blue: COR-A, dark blue: Roc-A) and green (light green COR-B, dark green: Roc-B) respectively. The model has been created from the crystal structure of the *C. tepidum* RocCOR construct (pdb: 3DPU). The missing Roc-B domain in the X-ray structure was modelled into a position analogous to Roc-A. Loop regions not resolved in the structural model were also modelled (see ‘Materials and Methods’ for details). (**c**) Sequence alignment and secondary structure assignment of the RocCOR tandem for *C. tepidum* Roco and human LRRK2. Conserved residues are shown in red (identical amino acids) and orange (similar amino acids). Positions where the Parkinson mutations addressed in the present study appear in LRRK2 are marked by grey boxes. Spin-label positions are indicated by yellow boxes.

The Roco family comprises large multi-domain proteins that are characterized by the presence of a Ras (rat sarcoma)-like GTP-binding (G) domain called Ras of complex proteins (Roc) that always occurs in tandem with a C-terminal of Roc (COR) domain [1–3]. Roco family proteins can be found in bacteria, plants and animals. Four Roco proteins are identified in vertebrates, called leucine-rich repeat (LRR) kinase 1 (LRRK1), LRRK2, death-associated protein kinase 1 (DAPK1) and malignant fibrous histiocytoma amplified sequences with leucine-rich tandem repeats (MASL). Human MASL has the simplest architecture that is also found in other metazoans, plants and prokaryotes. In these proteins, the RocCOR tandem is always preceded by an LRR domain (Figure 1a). The human proteins LRRK2 and LRRK1 have, in addition to the RocCOR tandem, an N-terminal LRR and C-terminal kinase domain. DAPK1, which is only found in metazoans, is characterized by the presence of a tumour-suppressor DAPKs domain. Despite the variation in architecture of the Roco proteins, previous studies suggest that the function and structure of the catalytic core is conserved [4].

The most prominent member is LRRK2 that has been found to be mutated and activated in individuals suffering from familial Parkinson's disease (PD, OMIM no. 168600). Previously we have shown that Roco proteins belong to the class of G-proteins activated by nt-dependent dimerization (GADs) [5,6]. This class includes the signal recognition particle (SRP) and its receptor (SR) [7], membrane fission and fusion proteins like dynamin [8] and atlastin [9], anti-viral dynamin-like proteins like human guanylate-binding protein 1 (hGBP1) [10], the Toc (translocon at the outer envelope membrane of chloroplasts) family of plant protein transporters [11], tRNA-modifying enzymes like MnmE (Methyl-amino(N)-Methyl modifying protein E) [12] and its human orthologue GTPBP3 [13] and cytoskeletal proteins of the septin family [14]. Despite the increasing interest in this class of proteins on grounds of the medical relevance of its members, they are, by far, not as well characterized as their ‘conventional’ counterparts like the members of the Ras superfamily. Conventional guanine nt-binding proteins (G proteins) like Ras cycle between a GDP- (‘off’) and a GTP-bound (‘on’) state with the help of regulatory proteins. GTPase-activating proteins (GAPs) complement and/or stabilize the active site to increase the rate of GTP hydrolysis by several orders of magnitude [15,16]. Nt exchange, i.e. release of GDP or GTP, on the other hand, is accelerated by interaction with guanine nt-exchange factors (GEFs), which strongly reduce nt affinity.

In contrast, GADs show reciprocal complementation of their active sites and seem not to require GAPs and GEFs, as they appear to contain the elements necessary for the nt-regulated switching cycle [5]. They exhibit low nt affinity, rendering the need for GEFs to exchange GDP for GTP unnecessary and dimerize upon GTP binding to supplement each other with elements needed for efficient GTP hydrolysis, rendering GAPs as accessory proteins obsolete. Although the basic principles mentioned above seem to apply to all GADs, significant mechanistic differences have been observed [5].

It has been a challenge to study the LRRK2 G-protein cycle [17]. A few GAPs and GEFs have been reported for LRRK2; however, none of these putative regulators directly bind to the Roc domain [18–20]. Furthermore, LRRK2 has a low nt affinity (micromolar range) and a hydrolysis rate similar to that of other Roco proteins and small GTPases [21,22]. Data of various studies suggest that LRRK2 forms, like bacterial Roco proteins, an active dimer via the COR domains [6,21,23,24]. Due to the lack of adequate amount of recombinant LRRK2 proteins, structural understanding has mainly come from work with related Roco proteins [17]. Crystal structures of the *Chlorobium tepidum* RocCOR (*Ct*RocCOR) unit [25] (Figure 1b) and *Methanosarcina barkeri* Roco2 RocCORΔC unit [6] reveal a typical small G-protein fold for the Roc domain. The COR domains in the *Ct*RocCOR structure are a dimer in which the N-termini interact with the, between man and bacteria highly conserved (Figure 1c), Roc domain of the same protomer and the less conserved C-termini function as a dimerization device [6,17,25]. Consistent with other GADs, dimerization is essential for GTPase activity; the Roco proteins in *C. tepidum* (Arg^543^; Figure 1c) and *M. barkeri* use an arginine finger of one monomer to complete the catalytic machinery of the other monomer (Arg^543^ in *C. tepidum*; Figure 1c). Together, these data thus suggest that Roco proteins, including LRRK2, belong to the GAD family of G-proteins. However, the *C. tepidum* and *M. barkeri* structures were only solved in the nt-free and GDP-bound states respectively [6,25]. Therefore, the exact mechanism of the Roc G-protein cycle is still not well understood.

Using a stable recombinant LRRK2 RocCOR–kinase fragment, we obtained more detailed kinetic data for the G-protein cycle which suggests that, in analogy to bacterial Roco proteins, dimerization is essential for efficient GTP hydrolysis. To gain insights into the solution structure and conformational dynamics of the RocCOR dimer, we investigated the *C. tepidum* RocCOR unit using site-directed spin labelling [26,27] and EPR spectroscopy. This technique has already been successfully applied to characterize relative motions and/or association/dissociation of the G domains in course of the GTPase cycle for several other proteins of the GAD family, like MnmE [12,28], Toc34 [29] and hGBP1 [30]. We focused on three major questions: (i) does the COR domain provide a stable scaffold for the Roc domains, (ii) what are the conformational dynamics of the Roc dimer in course of the GTPase cycle and (iii) how do mutations in the conserved Roc/COR interface influence these conformational dynamics?

## MATERIALS AND METHODS

### Protein expression, purification and GTP hydrolysis

The indicated cysteine mutants were generated by the method of Quick change. The *C. tepidum* RocCOR [amino acid (AA) 412–946] mutants were expressed and purified as previously described for the corresponding wild-type protein [21]. The LRRK2 Roc-COR-kinase (AA 1334–2147) fragments were expressed in *Sf*9 cells from a pfastBac vector containing an N-terminal histidine-tag (Invitrogen) and subsequently purified by affinity chromatography using Ni-NTA (nitrilotriacetic acid) matrix. A multiple turnover radioactive charcoal assay was used to measure the GTPase activity of the isolated LRRK2 Roc-COR-kinase mutants. For this, 100 nM of the mutants was incubated in buffer (50 mM NaCl, 20 mM Tris/HCl, 10 mM MgCl_2_, pH 7.5, 1 mM DTT, 0.5 mg/ml BSA) with up to 1 mM GTP including GTP-γ-^32^P at 25°C. Samples were taken at the indicated time points and immediately quenched with ice-cold 20 mM phosphoric acid containing 5% activated charcoal. The charcoal-bound non-hydrolysed GTP was precipitated by centrifugation and the supernatant containing organic phosphate was subsequently subjected to scintillation counting. The data were fitted by GraFit (Erithacus software).

### Spin labelling

The spin label (1-oxyl-2,2,5,5-tetramethylpyrroline-3-methyl) methanethiosulfonate (MTSSL; Enzo life sciences) was covalently attached to the cysteine residues of the RocCOR mutants. In brief, the protein in buffer (150 mM NaCl, 30 mM Tris/HCl, 5 mM MgCl_2_, pH 7.5) was incubated with 10 mM DTT for ∼12 h. DTT was removed by repeated buffer exchange using the same buffer. Afterwards, the protein was incubated for ∼12 h with 1 mM MTSSL and excess label was also removed by repeated buffer exchange. For EPR [double electron–electron resonance (DEER)] experiments at low temperature (50 K) the buffer was supplemented with 5% glycerol (v/v). We omitted the use of deuterated solvents (that are commonly used to slow down spin relaxation and to increase the accessible distance range and/or increase the signal-to-noise ratio) to safely exclude possible isotope effects on nt-binding and conformational changes and/or shifts of the conformational equilibrium induced by nt-binding. For the different nt-bound states either 1 mM GDP, 1mM 5′-guanylyl imidodiphosphate (GppNHp) or 1 mM GDP, 1 mM AlCl_3_ and 10 mM NaF was added respectively. Spin concentrations have been determined by double integration of room temperature continuous wave (cw) spectra and comparison with reference samples of known spin concentration and have been used to calculate spin-labelling efficiencies that have been found to vary significantly between the different RocCOR mutants (40%–100%). For all EPR experiments, the protein concentrations were 50–100 μM.

### EPR spectroscopy

CW EPR spectra were recorded at room temperature (298 K) with a home-made EPR spectrometer equipped with a Bruker dielectric resonator (MD5), with the microwave power set to 0.4–0.6 mW and B-field modulation amplitude adjusted to 0.15 mT. Samples were loaded into EPR glass capillaries (0.9 mm inner diameter, sample volume 20 μl).

DEER measurements were accomplished at X-band frequencies (9.3–9.4 GHz) with a Bruker Elexsys 580 spectrometer equipped with a Bruker Flexline split-ring resonator ER 4118XMS3 and a continuous flow helium cryostat ESR900 (Oxford Instruments) controlled by an Oxford Intelligent temperature controller ITC 503S. Measurements were performed using the four-pulse DEER sequence [31,32]:

$$\begin{array}{ccc} & & \pi /2\left(\nu_{\mathrm{obs}}\right)-\tau_{1}-\pi \left(\nu_{\mathrm{obs}}\right)-t^{\prime }-\pi \left(\nu_{\mathrm{pump}}\right)\\ & & \;-\left(\tau_{1}+\tau_{2}-t^{\prime }\right)-\pi \left(\nu_{\mathrm{obs}}\right)-\tau_{2}-\mathrm{echo} \end{array}$$

**Figure 2:**
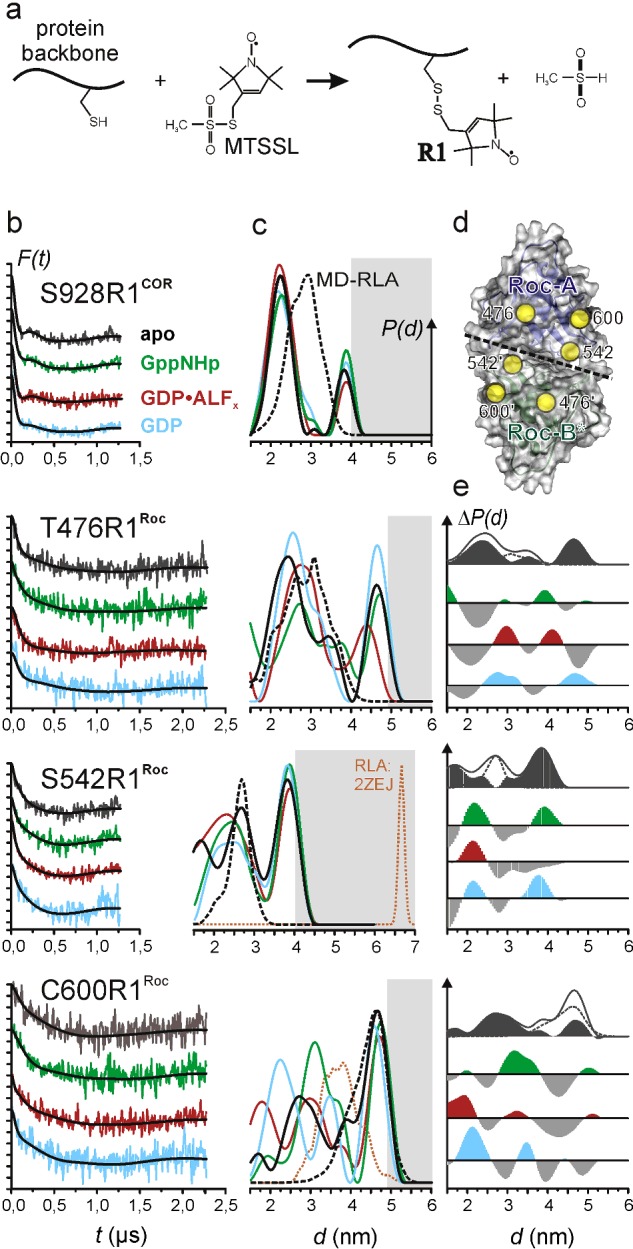
Interprotomer distances in *Ct*RocCOR (**a**) Site-directed spin labelling. After site-directed mutagenesis to replace the residue of interest by cysteine, reaction of the MTSSL with the thiol group of the cysteine yields the spin-label side chain commonly abbreviated as R1. (**b** and **c**) DEER data recorded at X band (9.3–9.4 GHz). (**b**) Background-corrected dipolar evolution data *F(t)*. Tick marks are separated by 0.05. (**c**) Distance distributions *P(d)* obtained by Tikhonov regularization (solid lines) and by MD-RLA (see text) of the dimer model shown in Figure 1 (**a**; black, dashed) or the dimer structure of LRRK2–Roc (pdb: 2ZEJ; orange, dashed). (**d**) Bottom view of the Roc dimer in the model (Figure 1 **a**), showing the locations of the label positions marked by spheres at the positions of their Cα atoms. The interface between the two Roc domains is marked by a dashed line. (**e**) Difference distance distributions Δ*P(d*). From top to bottom: *P(d)^apo^*–*P(d)^MD-RLA^, P(d)^GppNHp^*–*P(d)^apo^, P(d)^GDP-AlFx^*–*P(d)^apo^, P(d)^GDP^*–*P(d)^apo^*. The *P(d)^apo^*–*P(d)^MD-RLA^* plots are shown together with both *P(d)*s. Positive contributions in Δ*P(d)* for the nt-bound states are coloured according to the data in (**a**) and (**b**). Negative contributions are shown in grey. The difference amplitudes have been scaled for better visualization.

A two-step phase cycling [+(x),–(x)] was performed on π/2(*ν*_obs_). Time *t*′ is varied, whereas *τ*_1_ and *τ*_2_ are kept constant. The dipolar evolution time is given by *t*=*t*′–*τ*_1_. Data were analysed only for *t* > 0. The resonator was over-coupled to *Q* ≈ 100; the pump frequency *ν*_pump_ was set to the centre of the resonator dip and coincided with the maximum of the nitroxide EPR spectrum, whereas the observer frequency *ν*_obs_ was ∼65–75 MHz higher, coinciding with the low-field local maximum of the spectrum. All measurements were performed at a temperature of 50 K with observer pulse lengths of 16 ns for π/2 and 32 ns for π pulses and a pump pulse length of 12 ns. Proton modulation was averaged by adding traces at eight different *τ*_1_ values, starting at *τ*_1,0_=200 ns and incrementing by ∆*τ*_1_=8 ns. Data points were collected in 8-ns time steps. The total measurement time for each sample was 24–48 h. Data analysis was performed with the software package DeerAnalysis2013 [33] in the distance range 1.0–8.0 nm with regularization parameters according to the L-curve criterion. The mean distances given in Table 1 are calculated from the distance range 1.5–6.0 nm that is also shown in Figures 2 and 4.

**Table 1:** Theoretical and experimental inter-spin distances

	Experimental mean inter spin distance (nm)					
Mutant	apo	GppNHp	GDP•AlF_x_	GDP	Cβ–Cβ distance	Calculated (MD-RLA) mean distance (nm)
S928R1^COR^	2.61	2.68	2.51	2.59	2.5	2.82
	(2.24)*	(2.28)*	(2.23)*	(2.19)*		
T476R1^Roc^	3.19	3.35	3.21	3.39	2.3	2.84
+ L487A	2.75	3.24	2.40	2.74		
+ Y558A	3.26	3.01	3.07	3.10		
S542R1^Roc^	3.00	3.05	2.87	3.12	2.1	2.63
+ L487A	2.81	2.92	3.14	2.88		
+ Y558A	2.77	2.76	2.65	2.66		
C600R1^Roc^	3.74	3.73	3.46	3.37	3.5	4.42
+ L487A	3.15	3.12	2.79	3.35		
+ Y558A	2.95	2.74	2.41	2.64		

*Maximum of the major peak in the experimental distance distribution.

### Modelling of Roc-B and missing loop regions into the RocCOR dimer

Modelling of the full RocCOR dimer structure was performed using the software package YASARA Structure [34] with the following procedure: (i) The COR domain of a copy of RocCOR-A was overlayed on to Roc-B to create Roc-B in a position analogous to Roc-A. (ii) Internal missing loops in Roc-A were added using the ‘BuildLoop’ and ‘OptimizeLoop’ commands in YASARA, including 3–4 residues on both the N- and the C-terminal side of the gaps in the sequence. (iii) The initial Roc-B domain (without internal loops) was deleted and step (i) was repeated with RocCOR-A comprising the internal loops. (iv) For both RocCOR units, the connecting loops between Roc and COR were modelled as described in (ii). The YASARA scripts used for loop modelling are included in the supplementary materials.

### MD simulation and rotamer library analysis

The completed RocCOR dimer model (see above) was immersed in a water box [(128.9 Å)^3^; 1 Å=0.1 nm] filled with TIP3P water and ∼150 mM sodium and chloride ions, neutralizing the system's net charge. Periodic boundary conditions have been applied. Initial atomic clashes in the starting structure were removed by energy minimization (steepest descent). A 16 ns MD simulation was carried out in YASARA, utilizing the Amber03 force field [34] and using Particle Mesh Ewald (PME) summation for long-range electrostatic interactions with a cut-off at 7.86 Å. The time step for the calculation of intramolecular forces was 1.25 fs (simulation sub-step), intermolecular forces have been calculated every two simulation sub-steps (2.5 fs). The simulation temperature was 298 K. Temperature control was carried out by rescaling atom velocities. Pressure control was achieved by keeping the solvent (H_2_O) density at 0.997 g/ml and rescaling the simulation cell along all the three axes. Simulation snapshots have been taken each 83.3ps and analysed in YASARA. Total energies and mean backbone RMSD values compared with simulation time are shown in Supplementary Figure S2(a). RMSD and root-mean square fluctuations (RMSF) values per residue are shown in Supplementary Figure S2(b).

Inter-spin label distance distributions were simulated using a rotamer library of spin-labelled residues as described in [35]. The rotamer library implemented in the software package MMM2011 [35] consisted of 210 rotamers of MTSSL bound to cysteine, which have been used to replace the native residues at the positions of interest in the MD snapshots. Energies and resulting populations for individual rotamers were calculated by means of a Lennard–Jones potential at 175 K (the glass transition temperature for a water–glycerol mixture) and have been used as weights in the simulation of the distance distributions. For more details about the rotamer library analysis (RLA) see [35]. In total 33 structures from the trajectory have been subjected to RLA (at 0, 0.5, 1.0, …, 16.0 ns), summed up and normalized to obtain the final RLA distance distribution (MD-RLA).

## RESULTS

### DEER inter-spin distance determination

To follow structural changes in *Ct*RocCOR (RocCOR from now) that occur upon binding of different nts, we applied site-directed spin labelling. Positions mutated to cysteine for spin labelling with MTSSL, see Figure 2a) are Thr^476^ (close to the P-loop) and Ser^542^ (close to the switch II region) in the Roc domains. The native cysteine at position 600 in Roc was also used for labelling. Single-site labelling of RocCOR results in the introduction of two symmetry-related spin labels in the RocCOR dimer. Using a model of the *Ct*RocCOR dimer (Figure 1b), where Roc-B, missing in the X-ray structure [25] and unresolved loop regions have been modelled (see ‘Materials and Methods’), the Cβ–Cβ distances for these positions could be determined as 2.3 nm (Thr^476^), 2.1 nm (Ser^542^) and 3.5 nm (Cys^600^; Table 1). To verify the assumption that the COR dimer serves as a rigid scaffold for the two Roc domains and is not significantly influenced by Roc domain motions, a spin-label side chain was introduced replacing Ser^928^ at the ‘top’ of the COR domains (Cβ–Cβ: 2.5 nm). No significant impairment of GTPase activity by the mutations in comparison with wild-type could be observed (Supplementary Table S1).

We applied a pulsed EPR method, DEER or pulsed electron double resonance (PELDOR) [31,32], to measure distances between spin-label side chains ranging from 1.5 to 6 nm in frozen (50 K) samples. Figure 2 shows the results of the DEER measurements with RocCOR in four different states of the GTPase cycle; in the apo state without any nt (grey, black), in the active state with the non-hydrolysable GTP analogue GppNHp (green), in the GTP hydrolysis transition state (red) mimicked by GDP·AlF_x_ (GDP-aluminum fluoride) [36] and in the GDP-bound inactive state (blue). Figure 2(b) shows the background-corrected dipolar evolution data with fits obtained by Tikhonov regularization (see ‘Materials and Methods’) and Figure 2(c) the corresponding distance distributions. Mean distances calculated from the distance distributions are summarized in Table 1.

### The COR domain is a stable dimerization device

A spin-label side chain at position 928 at the ‘top’ of the COR dimer (Figure 1b) reports on the validity of the structural model and on possible conformational and dynamic changes upon binding of the different nts. In agreement with the presumed role of the COR domain dimer to function as a scaffold for the Roc G-domains, only minor changes are observed in the distance distributions represented by two broad peaks centred approximately 2.2 nm (∼70%–80% area) and 3.9 nm (∼20%–30%), indicating that no significant conformational changes upon binding of the different nts take place in the COR dimer (Table 1). The observed changes mainly concern the width of the short distance peak and the relative contribution of the second peak. The latter contributions in the distance distributions at ∼3.9 nm are at the upper boundary of the accessible distance range given by the dipolar evolution times. Nevertheless, validation of the DEER data analyses (Supplementary Figure S1) indicates that these peaks are significant, but also that their relative contributions partly depend on the background correction. Due to this ambiguity, we do not further discuss the observed changes of <10% in the relative contribution of this peak to the overall distance distributions.

We calculated inter-spin distance distributions that can be compared with the experimental results from the completed structural model (see ‘Materials and Methods’) for the RocCOR dimer applying a RLA approach [35] to account for the dynamics of the spin-label side chain (see ‘Materials and Methods’). To consider also small-scale protein backbone dynamics, we carried out a MD simulation (16 ns, Amber03 forcefield, PIP3P water, 298 K; Supplementary Figure S2) with the structural model and performed the RLA on snapshots equally distributed over the MD trajectory (MD-RLA; for details see ‘Materials and Methods’). The results of these analyses for the spin-labelled positions in RocCOR are shown as dotted lines (black) in the distance distributions in Figure 2(c). The MD-RLA distance distribution for S928R1 exhibits a broad peak centred approximately 2.8 nm, covering distances ranging from 2 to 4 nm. This is in line with the solvent-exposed location of S928R1 on the protein surface and inspection of the MD trajectory and the RLA distance distributions obtained for the single MD snapshots (Supplementary Figure S3) reveals that small-scale backbone dynamics not significantly contribute to the width of the calculated distance distribution [RMSF_Cα_< 0.2 nm for residue 928 and for the C-terminal half of the COR domain (residues 781-end); Supplementary Figure S2b]. Comparison with the experimental distance distribution (Figure 2b) shows clear deviations although approximately the same distance range is covered. The experimental distance distribution is bimodal and the major distance between the two spin-label side chains in the RocCOR dimer appears to be approximately 0.6 nm shorter than predicted from the MD-RLA. The latter observation most probably indicates a more tight arrangement of the COR dimer in solution, which could then also explain the presence of the long-distance peak: a conformation in solution different from the structural model might, due to increased steric hindrance of the spin label in a more closely packed COR dimer, cause two distinct rotamer populations with different orientations. Such differences between the X-ray model and the structure of the protein dimer in solution could possibly be explained by the observed crystal contacts that COR-A is involved in [25]. Alternative explanations for the bimodal character of the experimental distance distributions could be aggregation or multimerization of the protein under our experimental conditions or that a second stable conformation of the ROC C-terminal domain exists, which is responsible for the second peak in the experimental distance distribution. Although we cannot safely distinguish between these possibilities, in all cases the COR domains serve as a stable dimerization device as neither the conformation nor a possible two-state equilibrium is significantly influenced upon binding of the different nts.

### The Roc domains display conformational heterogeneity that prevails in the presence of different nts

The experimental distance distributions for all three spin-label mutations in the Roc domain (Figure 2) in the apo state are very broad and characterized by multiple peaks. Although the MD-RLA especially for positions 476 and 600 already predicts broad and multimodal distance distributions owing to their solvent-exposed location on the protein surface (Figure 2d), the exceptional experimental distribution widths, corresponding to the absence of clear dipolar modulations in the DEER form factors shown in Figure 2(b), indicate in addition conformational heterogeneity of the Roc domains. In particular, for T476R1 significant contributions at longer distances, for C600R1 at shorter distances and both for S542R1 are observed that do not coincide with the predictions from the structural model (Figure 1b), suggesting that the Roc domains in the model represent only one snapshot of the conformational ensemble that characterizes the G domains of the RocCOR tandem in solution. This observation is in line with the absence of electron density for the Roc-B domain in the crystal structure of the RocCOR tandem and further supports the notion that the Roc domains are generally highly mobile and that Roc-A is only visible due to stabilizing crystal contacts [25]. To identify additional conformational states, difference distance distributions [Δ*P(d)*] (Figure 2e) were calculated by subtracting the *P(d)* of the MD-RLA from the respective *P(d)* of the experimental data for the apo state. Grey-shaded areas indicate the remaining contributions of the distance distributions. It has to be noted that deconvolution of the experimental distance distributions is complicated due to the combined effects of spin-label rotamer distribution, protein conformational heterogeneity and experimental uncertainties. Furthermore, as discussed above for the DEER data for position 928, we cannot exclude a possible influence of background-correction artefacts. Nevertheless, the broadness and multimodality of the remaining contributions attributable to conformational states different from the crystal structure indicate the presence of at least two additional conformations. This is most obvious for position 542 where the difference distance distribution exhibits two clearly distinct populations. This leads us to the conclusion that the Roc domains in the RocCOR dimer in solution are characterized by at least three conformational states in equilibrium.

The distance distributions observed in the presence of GppNHp, GDP·AlF_x_ or GDP (Figure 2c) reveal changes upon nt binding, but the conformational heterogeneity observed in the apo state largely prevails under all conditions tested, again indicating the presence of multiple conformational states. To identify possible shifts in the occupancy of these states, difference distance distributions [Δ*P(d)*] (Figure 2e) were calculated by subtracting *P(d)* for the apo state from *P(d)* obtained in the presence of the respective nts. As an additional indication for the average direction of the inter-spin distance changes upon nt binding, we calculated the means of the experimental distance distributions (Table 1).

For S542R1, located directly in the dimer interface of the two Roc domains (Figure 2d), we observed only slightly increased mean distances upon binding of GppNHp (+0.05 nm) and GDP (+0.12 nm) and a slight decrease for GDP·AlF_x_ (–0.13 nm). Inspection of the distance distributions and Δ*P(d)* plots suggest that, bearing in mind the experimental error and the limitations of Tikhonov regularization, this can be explained by de-population of the conformational state characterized by the crystal structure and increased population of the conformational states characterized by distance distributions with mean distances of ∼ 2 and 4 nm respectively that have been identified in the *P(d)^apo^*–*P(d)^MD-RLA^* plots. Thus, our data show a clear influence of the bound nt on the occupancy of the conformational states. Remarkably, in the GppNHp- and GDP-bound states the population of both states increases, whereas in the GTP hydrolysis state only the state with short inter spin distances shows an increased population, resulting in the shortest mean inter-spin distance observed. Furthermore, the width of the short-distance peak, being significantly decreased compared with the distribution in the apo state in the 1.5–3.5 nm range, indicates reduced backbone and/or label dynamics, being in line with a fully assembled active site (bearing in mind that the putative arginine finger Arg^543^ is next to the label position) and thus a more rigid Roc dimer. Interestingly, in the GppNHp- and GDP-bound states, the width of the short-distance peak is even smaller than for GDP·AlF_x_, but the mean distances are ∼0.2 nm larger. This implies that nt binding, in general, causes reduced backbone and/or side chain dynamics at and near the nt-binding pocket.

The mean inter-spin distance for C600R1 (Table 1) also remains almost unaltered upon binding of GppNHp, but decreases in the presence of GDP·AlF_x_ (–0.28 nm) and GDP (–0.37 nm). This is also reflected in the Δ*P(d)* plots, which reveal increased population of conformational states characterized by shorter distances for the latter nts and population of a conformational state with inter-spin distances around the mean distance in the apo state. Nevertheless, for C600R1,as well as for T476R1, the correlation between the distance contributions in the *P(d)^apo^*–*P(d)^MD-RLA^* plots with the changes in population from the *P(d)^nt^–P(d)^apo^* plots is not as clear as for S542R1, possibly owing also to the increased signal-to-noise ratio for these datasets. The mean distances for T476R1 exhibit the smallest variation upon nt binding and the shortest mean inter-spin distance is observed in the apo state. This indicates that the Roc dimer does not show ‘open’ and ‘closed’ states like they have been observed for MnmE [12] in course of the GTPase cycle. More likely, the G domains remain largely associated and changes of their relative orientation and of specific secondary structure elements facilitate the structural requirements for the catalytic steps. Taken together, the results of the DEER inter-spin distance measurements suggest that binding of the different nts influences a complex equilibrium between multiple conformations of the Roc domains in the RocCOR dimer.

### PD mutations in the Roc domain cause reduced GTPase activity because of an altered nt-dependent G-domain conformational equilibrium

**Figure 3:**
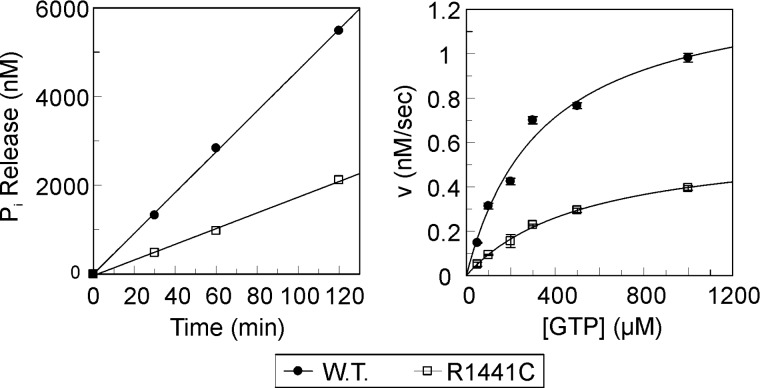
GTPase activity of human LRRK2 Roc-COR-kinase fragments Left: Inorganic phosphate release of 100 nM LRRK2 protein incubated with 500 μM γ^32^P-GTP during the indicated time. The slopes of the linear fits of phosphate release at different time-points yield the reaction rates (*v*). Right: The reaction rates (*v*) of wild-type and the R1441C PD-mutant of human LRRK2 Roc-COR-kinase proteins are plotted against substrate (GTP) concentration. The error bars show the standard error of the reaction rate calculated from at least three measurements. *k*_cat_ and *K*_m_ values are calculated by fitting the data with the Michaelis–Menten equation using GraFit (Erithacus Software).

The most prominent PD-mutations in the LRRK2 Roc domain are Arg^1441^ to cysteine, glycine or histidine. Previously it has been suggested that the LRRK2 R1441C mutation has reduced GTPase activity [37]. However, because of the lack of stable purified recombinant protein, it has been so far a challenge to obtain detailed quantitative data for the GTPase activity of LRRK2. We were able to express and purify small amounts of a stable LRRK2 fragment comprising the Roc-COR-kinase fragment from *Spodoptera frugiperda* Sf9 cells. A multiple turnover radioactive charcoal assay was used to measure the GTPase activity of the LRRK2 fragment. In this assay, the proteins are mixed with γ^32^P-labelled GTP and the subsequent P_i_ release is measured over time. The GTPase kinetics of the Roc-COR-kinase fragments of LRRK2 follow Michaelis-Menten kinetics. For the wild-type protein, its *k*_cat_ of phosphate release is 0.8 min^−1^ and the *K*_m_ of GTP is 343 μM. Consistent with the previous studies, the LRRK2 Roc (R1441C) PD-mutation results in a decreased GTPase activity (Figure 3) [21,37–40]. The *k*_cat_ of the R1441C mutant is ∼2-fold lower (0.37 min^−1^) than that of the wild-type protein, whereas the *K*_m_ value is ∼1.5-fold higher (541 μM) compared with wild-type. Unfortunately the quality and the amount of the purified LRRK2 Roc-COR-kinase fragment were not sufficient for detailed biophysical studies. Furthermore, the fragment contains 15 cysteines, thus making it a very difficult target for spin labelling with standard approaches.

**Figure 4:**
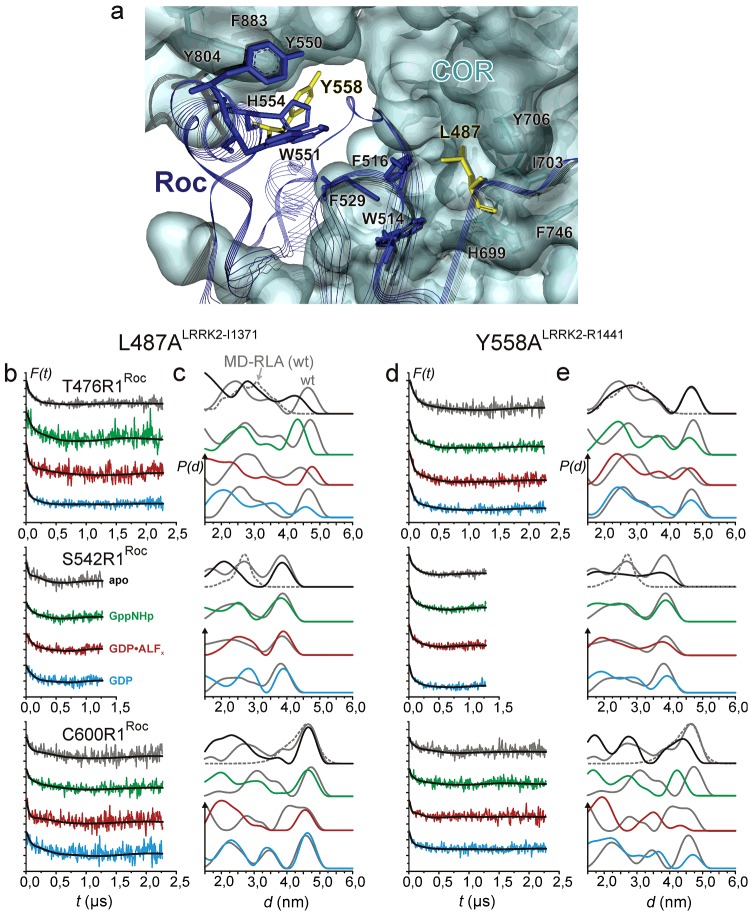
Influence of mutations in the Roc/COR interface on nt-dependent interprotomer distances in CtRocCOR (**a**) Atomic model showing the hydrophobic Roc-A/COR-A interface region. Residues and the transparent surface of COR-A are coloured in cyan. Roc-A residues and line ribbons are shown in blue. Residues Leu^487^ and Tyr^558^ (both in Roc-A) are coloured in yellow. (**b**–**e**) DEER data recorded at X band (9.3–9.4 GHz) for additional mutations (**b** and **c**) L487A (Ile^1371^ in LRRK2) and (**d** and **e**) Y558A (Arg^1441^ in LRRK2). (**a** and **c**) Background-corrected dipolar evolution data *F(t)*. Major tick marks are separated by 0.2. (**b** and **d**) Distance distributions obtained by Tikhonov regularization (solid lines). Grey distance distributions are for the respective spin-label mutants without additional mutations (Figure 2 **c**) and for the MD-RLA (dotted distributions) of the dimer model shown in Figure 1(**b**).

The position analogous to R^1441LRRK2^ in *Ct*RocCOR is Tyr^558^, whereas the less frequently PD-related mutation Ile^1371LRRK2^ corresponds to Leu^487^ *Ct*RocCOR (Figure 1c) [25]. Both Leu^487^ and Tyr^558^ are in close proximity in the hydrophobic interface between Roc and COR (Figure 4a). We previously have generated the mutants L487V, L487A and Y558A [25]. All mutant showed a strongly reduced GTPase activity, most probably due altered interaction between the Roc and the COR domains. Since the L487A and Y558A mutations had the largest impact on GTPase activity (∼40-fold reduction), we analysed the impact of these mutations on the structure and dynamics of the Roc domains in the apo and different nt-bound states (Figures 4b–4e).

Replacing Leu^487^ by alanine seems to hardly influence the Roc dimer interface in the absence of nts, as deduced from the almost unaltered distance distribution for S542R1, except reduced relative amplitude of the long distance peak. In contrast, significant alterations are observed for T476R1 and C600R1, mainly characterized by increased distance distribution widths and a shift towards shorter distances, as can also be seen from comparison of the mean inter-spin distances reported in Table 1. This suggests that the Roc dimer remains stable but appears to be detached from the COR scaffold, what might also be reflected in the increased flexibility observed for position C600R1. Upon binding of the different nts in most cases also a shift towards shorter distances is observed (Table 1) and the distance distributions are broader, further supporting the notion that the PD-mutation significantly weakens the Roc–COR interaction. This effect is even more pronounced in the Y558A mutant, where in contrast with L487A also the Roc dimer interface appears to be more strongly affected, as deduced from the broad and flat distance distribution ranging from <1.5 to 4.5 nm for S542R1 in the apo state. This significantly increased flexibility of the Roc dimer interface largely prevails in the nt-bound states, indicating that the ability of the Roc domains to adopt a functional dimer conformation, i.e. to populate the catalytically-active conformational state, is strongly impaired, thus explaining the reduced GTP hydrolysis rate. Similar effects on the distance distributions are observed for C600R1, whereas the T476R1 label senses only minor changes by the Y558A substitution, mainly characterized by an increased probability to find shorter distances. Thus, removing a single hydrophobic residue from the Roc/COR interface strongly influences the complex conformational equilibrium of the Roc domains. This indicates that the COR domain dimer functions not only as a scaffold to hold the Roc domains in place, but that it also permits fine-tuning of their structure and conformational dynamics for efficient GTP hydrolysis.

## DISCUSSION

The results of the DEER distance measurements show that the COR domains in the RocCOR tandem dimer serve as a scaffold for the Roc G-domains by forming a constitutive dimer through interaction of their C-terminal subdomains [6,25]. Furthermore, the *K*_m_ of GTP observed with the purified LRRK2 Roc-COR-kinase fragment (343μM) is comparable to that found for mouse full-length LRRK2 (210 μM) and for recombinant human LRRK2 Roc domain (553 μM) [21,22]. In contrast, the hydrolysis rate (*k*_cat_) of the recombinant Roc-COR-kinase fragment is ∼20-fold slower compared with homologous expressed mouse full-length LRRK2 (13.8 min^−1^), but 40-fold faster than that of monomeric recombinant human LRRK2 Roc domain (0.02 min^−1^) [21,22], suggesting that, in analogy to bacterial Roco proteins [6,25], dimerization is essential for efficient GTP hydrolysis.

Furthermore, our DEER data support the notion based on the absence of defined electron density for the second Roc domain in the RocCOR dimer and on proteolytic digestion experiments [21] that the Roc G-domains are highly mobile entities. They sample multiple conformations, one of which seems to be represented by the *Ct*RocCOR crystal structure. Given the large experimentally observed distance distribution widths of ∼3 nm for all positions and under all conditions tested in the present study, the theoretical mean distances lie well within these distance intervals, as can be seen from the difference distance distributions in Figure 2(e) and the mean distance values reported in Table 1. A swapped dimer, where the N-terminus of one Roc domain interacts with the C-terminus of its counterpart, as it has been found for isolated LRRK2–Roc [41], appears unlikely for *Ct*RocCOR, as comparison with theoretical distance distributions calculated from the LRRK2–Roc dimer structure (Figure 2c, orange, dashed distributions) reveals striking disagreement especially for position 1421 in LRRK2 corresponding to Ser^542^ in *Ct*RocCOR. We cannot exclude that the conformational equilibrium in which the Roc domains are involved might involve states that resemble the swapped dimer structure, as the expected inter-spin distance of ∼6.7 nm is beyond the detection limit of the DEER experiment. Nevertheless, available crystal structures of RocCOR tandems reveal that formation of a swapped Roc dimer would lead to serious clashes with the N-terminal part of the respective COR domain [6,25]. We further cannot exclude that the human Roc protein and the bacterial Roco protein exhibit significant structural differences. However, taking into account the high conservation between *Ct*RocCOR and LRRK2 of especially the region around Switch II, where Ser^542^ is located, our results strongly support for the notion that the swapped-dimer structure has no functional relevance [17,21]. Our data also show that binding of different nts leads to complex alterations of the occupancies of the conformational states. The distance measurements further show that no large-scale G domain motions take place during the GTPase cycle, like they have been observed by EPR and/or FRET distance measurements for other members of the GAD family. We previously showed that in the constitutive dimer of the tRNA-modifying enzyme MnmE the G domains in the apo and GDP-bound state adopt an ‘open’ conformation where they are separated by ∼2–3 nm and that binding of GTP and subsequent hydrolysis leads to a ‘closed’ conformation where also the G-domains dimerize [12,28]. We observed a similar mechanism for hGBP1, which is a monomer in the apo and GDP-bound states, but binding of GTP induces dimerization of the G domains, leading to conformational changes in neighbouring domains that further tighten the dimer interaction [30]. A slightly different mechanism was recently observed for the Toc34 GTPase homodimer involved in chloroplast pre-protein translocation [29]. In this case, the GTP bound state was found to exhibit an ‘open’ and very dynamic conformation, whereas the G-domains in the GDP- and GDP·AlF_x_-bound states form a quite rigid (‘closed’) dimer. In contrast with that, we could not identify ‘open’ and ‘closed’ conformations for the G-domains in the RocCOR dimer. Our observations indicate that the Roc domains remain associated throughout the whole GTP hydrolysis cycle, as can be seen from comparison with the mean inter-spin distances reported in Table 1. Nevertheless, in the GTP hydrolysis transition state mimicked with GDP·AlF_x_ the mean inter-spin distances and distribution widths especially for S542R1 appear to be decreased, suggesting a more rigid assembly of the G domains compared with the other states. Thus, mutual complementation of the G-domain's catalytic centres by protrusion of the in prokaryotes conserved arginine finger (Arg^543^) into the active site of the other protomer to promote GTP hydrolysis would take place in RocCOR via subtle domain movements, e.g. relative rotation of the two domains and local conformational changes in the Roc domains rather than by large scale conformational changes and domain association. Furthermore, these observations indicate that, in contrast with other GADs like MnmE and hGBP1 where the isolated G domains suffice to form a functional dimer, the COR dimer scaffold is not only necessary to keep the Roc G-domains in close vicinity, but also for fine-tuning of their conformational equilibrium to favour states that are competent for nt-exchange and GTP hydrolysis respectively. Consequently, our findings also provide a rationale for regulation of the GTPase activity by neighbouring domains in Roco proteins.

We confirmed that the LRRK2–R1441C PD-related mutation results in decreased GTPase activity. The *C. tepidum* RocCOR structure revealed that the position analogous to this LRRK2 PD-mutation site is located in the hydrophobic Roc/COR interface that is highly conserved between bacteria and man [25]. We showed that mutations in the *Ct*Roc domain that are located in the Roc/COR interface and that have been shown to have the strongest effects on GTPase activity [25] significantly alter the conformational equilibrium of the G-domains, mainly reflected in increased flexibility and a bias towards (additional) conformations characterized by shorter inter spin distances, especially in the nt-bound states. Consistently, previous data suggest that PD-mutations in the LRRK2 Roc domain de-stabilize the protein, whereas LRRK2 PD-mutations in the COR domain alter the interaction between the Roc and COR domain [42,43]. This further supports the notion that the interaction between the N-terminal half of the COR domain and Roc controls the conformational equilibrium of the G domains and that perturbations by mutations in this interface largely alter their energy landscape, disfavouring population of the catalytically-active conformational states.

Although we cannot exclude that the LRRK2 RocCOR domain has a different structure and activation mechanism than bacterial Roco proteins, it seems rather unlikely. Recent data have shown that LRRK2 has a similar low nt affinity and hydrolysis activity to that of the bacterial Roco proteins [21,22]. Like bacterial Roco proteins, LRRK2 forms an active dimer via the COR domains and our data suggest that GTPase activity depends on dimerization [6,21,23,24]. The PD mutations in the LRRK2 RocCOR domain, as well as the PD-analogous mutations in *Ct*RocCOR, do not affect nt binding, but do results in impaired GTPase activity [25,37,38]. We therefore would like to postulate that PD-related mutations in the conserved Roc/COR interface of both LRRK2 and *C. tepidum* have a strong effect on the dynamics of the Roc domains and that this is the primary cause of the decreased GTPase activity of both proteins [25,37–40].

## Abbreviations

AlFx: aluminum fluoride
COR: C-terminal of Roc
*Ct*RocCOR: *Chlorobium tepidum* RocCOR
cw: continuous wave
DAPK1: death-associated protein kinase 1
DEER: double electron–electron resonance
GAD: G-proteins activated by nt-dependent dimerization
GAP: GTPase-activating protein
GEF: guanine nt-exchange factor
hGBP1: human guanylate-binding protein 1
LRR: leucine-rich repeat
LRRK: leucine-rich repeat kinase
MASL: malignant fibrous histiocytoma amplified sequences with leucine-rich tandem repeats
MnmE: Methyl-amino(N)-Methyl modifying protein E
MTSSL: (1-oxyl-2,2,5,5-tetramethylpyrroline-3-methyl) methanethiosulfonate spin label
nt: nucleotide
PD: Parkinson's disease
Ras: rat sarcoma
RLA: rotamer library analysis
RMSF: root-mean square fluctuations
Roc: Ras of complex proteins
Roco: Ras of complex proteins (Roc) with a C-terminal of Roc domain
Toc: translocon at the outer envelope membrane of chloroplasts
